# An endogenous artificial microRNA system for unraveling the function of root endosymbioses related genes in *Medicago truncatula*

**DOI:** 10.1186/1471-2229-13-82

**Published:** 2013-05-16

**Authors:** Emanuel A Devers, Julia Teply, Armin Reinert, Nicole Gaude, Franziska Krajinski

**Affiliations:** 1 Max Planck Institute of Molecular Plant Physiology, Am Muehlenberg 1 14476, Potsdam (OT) Golm, Germany; 2 Swiss Federal Institute of Technology Zurich, Department of Biology, Zurich, Switzerland

## Abstract

**Background:**

Legumes have the unique capacity to undergo two important root endosymbioses: the root nodule symbiosis and the arbuscular mycorrhizal symbiosis. *Medicago truncatula* is widely used to unravel the functions of genes during these root symbioses. Here we describe the development of an artificial microRNA (amiR)-mediated gene silencing system for *M. truncatula* roots.

**Results:**

The endogenous microRNA (miR) mtr-miR159b was selected as a backbone molecule for driving amiR expression. Heterologous expression of mtr-miR159b-amiR constructs in tobacco showed that the backbone is functional and mediates an efficient gene silencing. amiR-mediated silencing of a visible marker was also effective after root transformation of *M. truncatula* constitutively expressing the visible marker. Most importantly, we applied the novel amiR system to shed light on the function of a putative transcription factor, *MtErf*1, which was strongly induced in arbuscule-containing cells during mycorrhizal symbiosis. MtPt4 promoter driven amiR-silencing led to strongly decreased transcript levels and deformed, non-fully truncated arbuscules indicating that *MtErf*1 is required for arbuscule development.

**Conclusions:**

The endogenous amiR system demonstrated here presents a novel and highly efficient tool to unravel gene functions during root endosymbioses.

## Background

In the past decades, legumes have been established as important model systems to discover the molecular and physiological background of the root nodule and arbuscular mycorrhizal symbiosis. Analysis of gene function during root endosymbioses requires reverse genetics approaches based on expression perturbation experiments. In the past, RNA interference (RNAi) or virus induced gene silencing (VIGS) has been widely applied to produce plant knock-down mutants. Both systems exploit endogenous posttranscriptional gene silencing (PTGS) pathways of eukaryotes [1-7].

An efficient VIGS system has not yet been established for *M. truncatula,* hence RNAi approaches have been widely applied to elucidate gene functions in *Agrobacterium rhizogenes* transformed roots. However, previous knock-down approaches in this system using RNAi constructs often did not lead to consistent results due off-target effects of RNAi approaches. RNAi is based on a hairpin construct with short inverted sequence fragments of the gene of interest separated by an intron and is processed via the IR-PTGS pathway. The expressed RNA folds into a perfect matched double strand and is processed by DCL4 to short interfering RNAs (siRNAs). However, in some cases the approach is limited by inefficient knock down of the target gene in legumes due to unknown causes [8]. Additionally, the RNAi approach leads to heterogeneous accumulation of siRNA products, derived from the expressed hairpin which can lead to unspecific downregulation of related genes (off-targets), especially in large gene families with high sequence similarity [9]. Also, a mechanism called transitivity leads to an amplification and spreading of the siRNA species, yielding secondary siRNAs independent of the primary siRNA signal [10]. These secondary siRNAs cover sequence information outside of the designed RNAi construct, thus enhancing off-target effects. There is precedent for artificial miRNAs to be more specific as RNAi constructs [11,12], here we suggest artificial miRNAs as an alternative tool for gene knock down approaches. However, we do not provide a direct comparison of both approaches with regard to efficiency and target specificity.

Analyzing gene functions by gene knock out approaches in *A. rhizogenes* transformed root systems is also hampered by a high variability within the experimental system with independent transformation events being present in a root system after *A. rhizogenes* transformation. Hence, to facilitate investigating gene functions in non-uniformly transformed root systems, a strong expression strength of the gene knock down constructs is required. However, the widely applied 35S promoter for driving knock down constructs mediates a rather weak expression strength in *M. truncatula* roots [13], with particularly weak expression in arbuscule-containing cells of mycorrhizal roots [14]. We therefore developed a vector series with three different promoters for knock down construct expression, either the 35S promoter or the ubiquitin 3 promoter of *Arabidopsis thaliana* or the MtPt4 promoter of *M. truncatula*. The latter is mediating a particular strong expression in arbuscule-containing cells [15].

Arbuscules are intracellular fungal structures formed in the plant’s inner cortical cell layers. The development of arbuscules requires a profound reprogramming of the root cell [16], and a wide number of genes which are specifically expressed in arbuscule containing cells have been identified [17,18]. However, an analysis of the precise role of these genes during arbuscule development and function is often hampered by the previously mentioned inconveniences regarding expression perturbation experiments in mycorrhizal *M. truncatula* roots.

Here we demonstrate that mtr-miR159b is effectively processed from its precursor molecule and thus represents a highly suitable backbone for the expression of amiRs in *M. truncatula*. Efficient target gene knock-down could be validated by an amiR against a visible marker in an heterologous system and in *M. truncatula*. Additionally, we used the MtPt4 promoter, which mediates a strong expression in mycorrhizal roots [15] for driving the expression of an amiR against a previously identified putative transcription factor (*MtErf*1). Knock-down of *MtErf*1 expression resulted in reduced expression of levels of *Rhizophagus irregularis* genes indicating reduced mycorrhizal colonization. Moreover, *MtErf*1 seemed to be required for arbuscule development, since only truncated, non-fully branched arbuscules were present in roots with amiR-silenced *MtErf*1 expression.

## Results and discussion

### miR159b represents a suitable backbone for artificial microRNA (amiR) expression in *M. truncatula*

**Figure 1:**
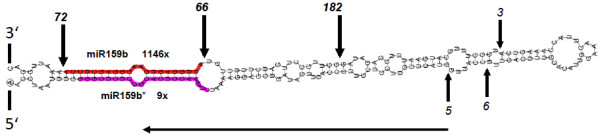
**mtr-miR159b represents a suitable backbone for amiR expression in *M. truncatula.*** Vertical arrows and numbers indicate the exact positions of degradome tags. The horizontal arrow indicates a loop-to-stem DCL1 processing. The mature miR159b is labeled in red, the miR159b* is labeled in purple. The read numbers of miR159 and miR159* from the small RNA libraries [23] are given.

Expression of artificial miRNAs requires a miRNA backbone sequence, of which the mature miRNA is replaced by an artificial miRNA (amiR), which binds and cleaves its target sequence(s). One prerequisite for amiR constructs is that star sequences and other small RNAs deriving from these constructs do not accumulate, and therefore could not regulate other off-targets. For correct processing of the amiR, the endogenous miRNA backbone must have a non-canonical loop-to-base processing type, with the first cleavage step, which seems to be most critical for miRNA processing occurring in the top region of the precursor independent of the miRNA sequence itself [19,20]. It is assumed that a clear physical separation of the first cleavage position and the miRNA sequence provides a high flexibility for manipulation of amiR sequences [19,21]. A previous study employed the *A. thaliana* loop-to-base processed miR319 as precursor for amiR expression in *M. truncatula*, which led to a significant downregulation of flottilin gene expression in roots [22]. However, in order to optimize expression of amiRs in *M. truncatula*, we decided to use an endogenous miRNA molecule as precursor. For this purpose, we screened our recent miRNA and degradome data of *M. truncatula* roots [23] for amiR backbone sequences and selected mtr-miR159b as a suitable precursor (Figure 1) since it showed all the necessary features mentioned above. The distribution of degradome tags across the miR159b precursor sequence confirmed the loop to base processing for this miR159 family member in *M. truncatula*. The miR159b primary transcript sequence was cloned in the pBluescript II SK+ vector flanked by restriction sites, which allow a directed cloning of the amiR sequences in the appropriate binary vectors for plant transformation (Additional file 1: Figure S1.)

### A vector system for expression perturbation experiments by *A. rhizogenes* mediated root transformation

For reverse genetic approaches in *M. truncatula* roots, we developed a vector series (pRed), where a constitutively expressed dsRED gene allows the easy detection of transformed roots (Additional file 1: Figure S2). We have developed expression vectors (pRed-Exp) and vectors for RNAi (pRed-RNAi). Both types of vectors are available with three different promoters for expression of the gene or RNAi construct, namely the 2×35S promoter, the ubi3 promoter of *Arabidopsis thaliana* and the MtPt4 promoter of *M. truncatula*[15].

### The miR159b-mediated amiR expression mediates strong silencing in tobacco leaves

**Figure 2:**
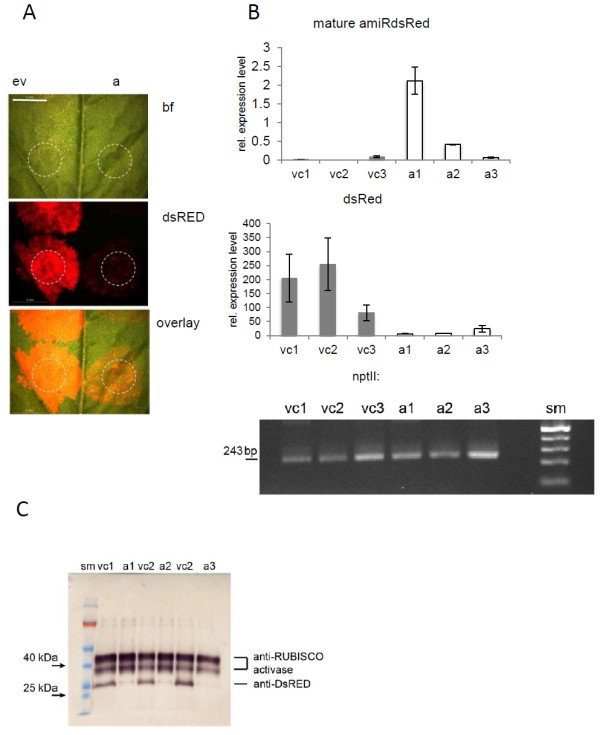
**The mt-miR159b-mediated amiR-silencing is efficient in tobacco leaves. A**) DsRED fluorescence is strongly reduced after infiltration of the pRed-35Spro::amiR-*dsred* (**A**) construct (a) as compared to empty vectors (ev). Bright filed image (bf), DsRED fluorescence (dsRED) and an overlay of both channels are shown. Infiltration site are indicated by dotted lines. Scale bar: 5 mm. **B**) Relative levels of mature amiR-dsred determined by stem loop qRT-PCR, relative transcript levels of dsred measured by qRT-PCR and transcript levels of *npt*II (kanamycin resistance) are shown. vc: vector controls, a: amiR samples sm: size marker, amplicon size of 243 bp is indicated. **C**) Western blot demonstrating reduced expression of dsRED after amiR-dsred mediated silencing of dsRED in tobacco leaves. Shown are three replicates of amiR-silencing (a) and vector controls (vc) represented by proteins of three different infiltration spots on the same leaf, for each construct. Reduced expression of dsRED after amiR-dsred infiltration was visualized using an anti-dsRED antibody. Equal sample loading is shown by anti-RUBISCO activase.

To test the functionality of the mtr-miR159b backbone system, we first generated amiRs targeting dsRED and tested them in a heterologous system using dsRED fluorescence as visible marker for gene silencing. An amiR against dsRED was designed using the WMD3 web microRNA designer. Constructs were generated using an overlapping PCR strategy as recently described [12], where the mature miR159b sequence was replaced by sequences complementary to a selected region of the target gene. The novel star sequence was designed in a way that the secondary structures of the miR159b backbone were conserved. In a first attempt to test the mtr-miR159b-mediated amiR expression, we introduced 35S_pro_::amiR-*dsred* constructs by agroinfiltration in tobacco (*Nicotiana benthamiana*). Since the vector used for transformation (pRed-35S_pro_::amiR-dsred) carried a constitutively expressed dsREDvisible marker for transformation, we expected that the amiR-dsred expression from the same vector will mediate a silencing of the dsRED. As a control we transformed the same leaves with an empty pRed-35S_pro_ vector. As expected, infiltration sites of the empty vector control showed a dsRED fluorescence, whereas infiltration of the pRed-35S_pro_::amiR-dsred construct led to only weak fluorescence (Figure 2). Analysis of the mRNA levels isolated from the infiltration sites confirmed gene silencing by the amiR-dsred. The abundance of dsRed mRNA was significantly lower in all three amiR-infiltrated leaves as compared to the vector controls (Figure 2B). Also a Western blot confirmed the significantly lower accumulation of dsRED proteins in amiR-infiltrated leaves as compared to the vector controls (Figure 2C).

### The miR159b backbone driven amiR constructs lead to efficient knock-down in *M. truncatula* roots

**Figure 3:**
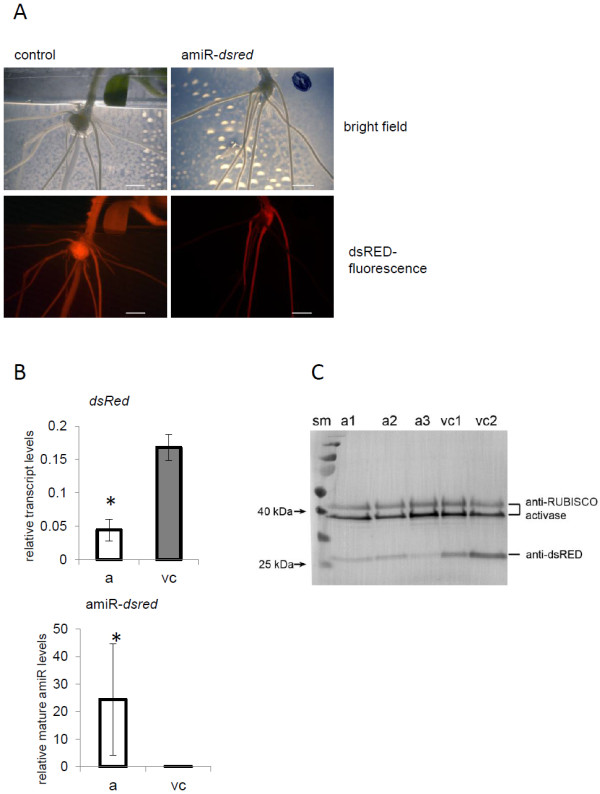
**Artificial microRNA (amiR)-mediated silencing of DsRED expression in *M. truncatula *roots. A**) Stable dsRED-expressing *M. truncatula* plants (35Spro::dsRED) were used for *A. rhizogenes* mediated root transformation. After transformation with the amiR-*dsRed* construct (right), parts of the roots lost dsRED fluorescence due to amiR-mediated gene silencing. Scale bar represents 5 mm. **B**) Relative levels of mature amiR-dsred determined by stem loop qRT-PCR and relative transcript levels of dsred measured by qRT-PCR as determined by qRT-PCR. Values shown are mean values of 3–4 biological replicates +/− standard deviation. Asterisks indicate statistical differences (P<0.05). **C**) Western blot analysis confirmed reduced accumulation of dsRED proteins in roots after transformation with the amiR-*dsRed* construct (a). Shown are three replicates of amiR-silenced root samples and two vector controls (v). Reduced accumulation of dsRED protein after amiR-*dsred* infiltration were visualized using an anti-dsRED antibody. Equal sample loading is shown by anti-RUBISCO activase.

Next, we tested the functionality of miR159b-mediated amiR expression in roots of *M. truncatula*. Stably transformed *M. truncatula* plants, which constitutively expressed dsRED (ubi10_pro_::*dsred*) were used for this purpose. We expected a loss of dsRED expression in roots after transformation with amiR-*dsred* construct, which would indicate that the miR159b backbone-driven amiR system works in *M. truncatula* roots. *DsRed*-specific amiRs were cloned behind the pORE-E4 vector [24]. As control, empty vectors were used. As expected, root transformation with the amiR-*dsred* construct led to the growth of non-fluorescent roots, whereas in the vector controls all of the roots kept the dsRED fluorescence (Figure 3).

### MtPt4 promoter driven amiR silencing of *MtErf*1 points to a role of this TF in arbuscule-development

Finally we wanted to confirm that amiR-mediated gene silencing also works efficiently in arbuscule-containing cells. Since the 35S promoter seems to be only weakly active in arbuscule-containing cells [14], we used the MtPt4 promoter of *M. truncatula*[15], to enable a strong and specific expression of the amiRs. MtPt4 encodes for a phosphate transporter, which is strongly induced in arbuscule-containing cells [15,25].

**Figure 4:**
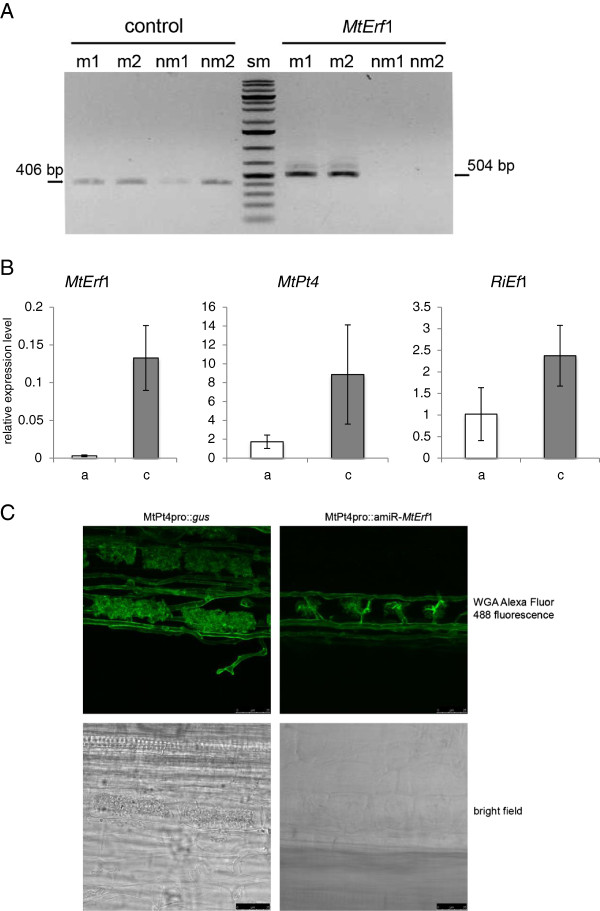
**Artificial microRNA (amiR)-mediated silencing of DsRED expression in *M. truncatula *roots. A**) *MtErf*1 transcripts are specifically induced in mycorrhizal roots (myc) and non- detectable in non-mycorrhizal (nm) roots. *MtErf*1 and *MtEf*1 as reference (control) transcripts were amplified from cDNA of mycorrhizal and non-mycorrhizal roots of *M. truncatula* by RT-PCR. Amplicon sizes are indicated. Two biological replicates are shown. **B**) Relative levels of *MtErf*1, *MtPt*4 and *R. irregularis* elongation factor 1 alpha (*RiEf*1) [29] were determined by qRT-PCR. Values shown are means of 3–4 biological replicates +/− one standard deviation. Each replicate consisted of several roots (n<5) transformed with either the MtPt4pro::amiR-*MtErf*1 (a) construct or MtPt4pro::*uid*A control (c). **C**) amiR-mediated *MtErf*1 knock-down results in deformed arbuscules. Shown are representative figures of arbuscules observed in roots transformed with either the MtPt4pro:: amiR-*MtErf*1 construct or the MtPt4pro::*uid*A control. Bright field images and WGA-Alexafluor 488 fluorescence are shown. Scale bar reesents 25 μm.

As a candidate gene for the proof of concept experiment we selected MtErf1, a putative member of the AP2 (APETALA2)-EREBP (ethylene-responsive element binding protein) transcription factor family. MtErf1 (medtr7g009410) was found to be strongly and specifically expressed in arbuscule-containing cells (Figure 4A and Additional file 1: Figure S3). The gene was previously identified by *M. truncatula* transcription factor (TF) profiling [26] in order to identify TFs, which are induced in mycorrhizal roots (Reinert et al. unpublished). MtERF1 consists of 392 amino acids with two AP2 domains in the N-terminal part of the protein. Hence, MtERF1 belongs to the subfamily of AP2 genes within the AP2-EREBP family. This is in contrast to previously described *M. truncatula* ERF transcription factors required for nod factor signal transduction [27,28]. It is worth mentioning, that the C-terminal part of the MtERF1 amino acid sequence seems to be only weakly conserved. However, homologous sequences were found in several plant species but not in the non-mycorrhizal plant *A. thaliana* (Additional file 1: Figure S4) supporting the assumption that MtERF1 might have specific functions in arbuscule development or functioning. To test this assumption, an *MtErf*1-specifc amiR was designed as described before and cloned behind the MtPt4 promoter of pRed. As a control, a MtPt4_pro_::*uid*A construct was used in the same vector. Roots that were transformed with the amiR construct, showed a significantly decreased expression of *MtErf*1 transcripts (Figure 4B). In addition, transcript levels of two diagnostic markers of a functional AM symbiosis, *MtPt*4 and *R. irregularis* elongation factor, were also clearly reduced in roots after amiR expression. This indicates that the MtPt4 promoter mediated an efficient amiR-silencing in arbuscule-containing cells. Moreover, reduced *MtErf*1 transcripts levels led to reduced expression of genes related to a functional mycorrhizal colonization and thus a lower level of mycorrhizal colonization. We next investigated the arbuscule morphology in vector control and amiR-*MtErf*1 roots. Arbuscule morphology was strongly impaired in amiR-*MtErf*1 roots with only small, truncated arbuscules being present in these roots (Figure 4C). This points to a function of this putative TF in arbuscule development and indicates the MtErf1 seems to be required for a full maturation of arbuscules.

## Conclusions

The endogenous amiR-mediated gene silencing system presented here provides a useful tool to investigate the function of genes involved in root endosymbioses. In addition, we showed that the MtPt4 promoter provides a strong expression of amiR constructs in mycorrhizal roots. AmiR-silencing of a putative transcription factor *MrErf*1 indicated a putative function of this gene during arbuscule development, since only defective arbuscules were observed in roots with reduced *MtErf*1 expression due to amiR-mediated gene silencing.

## Methods

### Biological Material

The following plants were used in this study: *Nicotiana benthaminana* cv. TW16, *Medicago truncatula* cv. Jemalong (A17) and *Medicago truncatula* cv. 2HA stably transformed with pKDSR(I) (see vectors and cloning procedures for vector details). Bacterial strains include *Escherichia coli* TOP10 (Invitrogen) or DH5α for cloning purposes, *Agrobacterium rhizogenes* strain ARqua1 [30] for Medicago root transformations and *Agrobacterium tumefaciens* strain GV2260 [31] for the leaf infiltration assay. The fungus *Rhizophagus irregularis* (strain BB-E, provided by Agrauxine, France) was propagated and used for mycorrhizal colonization studies as described in [23].

### *Medicago truncatula* growth, transformation and inoculation

*Medicago truncatula* seeds were scarified using concentrated H_2_SO_4_ and subsequently sterilized with HClO. The seeds were laid on a water agar plate and kept at 4°C in the dark overnight to synchronize the germination. Afterwards, the seeds were transferred to room temperature but remained in the dark for two days. *M. truncatula* root transformation was carried out with a modified protocol according to [32]. Seedlings were subsequently transferred to a fresh water agar plate, kept in the dark and incubated at room temperature for a further two days. Finally the seedlings were transferred into vertical square plates (amiRdsRED) or jars (amiR*MtErf*1) containing Fahraeus medium plus 25 μg/ml kanamycin. The plates and jars were kept in a phytotron for three weeks to allow the growth of transformed roots. The phytotron conditions were: 200 μE·m^-2^·s^-1^, 16 h/8 h day-night cycle, 22°C and 65% humidity. After three weeks the plants expressing the amiR- and control construct indicated by a DsRed fluorescence, were potted in a soil mix of quartz sand (0.6–1.2 mm)/expanded clay/vermiculite/inoculum mix (7:1:1 [v/v]). All potted plants were fertilized twice a week with 20 μM P_i_ Hoagland’s solution [33].

*M. truncatula* Jemalong, genotype 2HA was used for plant transformation with the vector pKDSR(I) (see vectors) [34].

### Leaf infiltration assays

*Nicotiana benthamiana* plants were grown for 4 weeks in a phytotron and used for the infiltration prior to the flowering phase. The experiment was repeated on two individual plants and six infiltrations each. Prior to infiltration, the plants were watered for 3h until the soil was water saturated. The leaves were infiltrated either with *Agrobacterium tumefaciens* (GV2260) containing pRed-35S_pro_::amiR-dsred or empty pRed-Expr. The bacteria were harvested, washed with AS-media (10 mM MES, 10 mM MgCl_2_, pH 5.6), resuspended to an OD_600_ of 0.8 with AS-media containing 150 μM acetosyringone and incubated for 3 h at room temperature on a shaker (50 rpm). Using a syringe, 500 μl of the bacterial suspension was infiltrated into the abaxial side of each leaf. Infiltration boarders were marked with a permanent marker. The plants were placed into a phytotron and analyzed after two and three days. After analysis, the marked leaf areas were excised, frozen in liquid nitrogen and stored at −80°C for further protein and RNA extraction.

### Staining and determination of fungal structures

Fungal structures were visualized with wheat germ agglutinin (WGA) conjugated with Alexa Fluor 488 (Invitrogen). In short, the approx. 1 cm long root sections were submerged for 5 min in 90°C hot 10% KOH [w/v] and washed five times with phosphate buffered saline (PBS) buffer (pH 7.4). The roots were then incubated overnight in PBS buffer (pH 7.4) containing 0.01% WGA Alexa Fluor 488 [w/v].

### Fluorescence imaging

The DsRed fluorescence of tobacco leaves and the roots of chimeric *M. truncatula* plants were monitored using a stereo-fluorescence microscope Leica M165 FC with a DsRed filter system (Leica 10447227). The exposure time for fluorescence was 500 ms and the gain setting was 2.0×. Overlay images were produced using Leica LAS-AF Version 2.8.1.

Confocal Images of WGA-Alexa Fluor stained arbuscules were collected on a Leica TCS-SP5 confocal microscope (Leica Microsystems, Exton, PA USA) using a 63× water immersion objective NA 1.2, zoom 1.6. Alexa Fluor was excited at 488 nm and emitted light was collected from 505 to 582 nm. Optical sections were acquired at 0.3–0.5 μm intervals. Images were processed using ImageJ software (Wayne Rasband, National Institutes of Health, USA; http://imagej.nih.gov/ij).

### Mapping of small RNA and degradome reads to miRNA precursors and design of artificial miRNAs

All *M. truncatula* precursor sequences belonging to the families of miR159 and miR319, were gathered and analyzed for small RNA and degradome read location as well as abundance. Small RNA and degradome reads were previously mapped to the Medicago 3.0 genome [23]. The information of the precise location of the reads and their abundance was manually annotated to the appropriate precursor. The 21 nt long artificial miRNA sequences against *dsRED* and *MtErf*1 were designed using the web miRNA designer WMD3 (http://wmd3.weigelworld.org/cgi-bin/webapp.cgi) following the instructions given on the website. Only green flagged sequences were chosen and checked against their target sequence and the Medicago genome v3.5 using psRNAtarget (http://plantgrn.noble.org/psRNATarget/). The sequence showing no or the least possible off-targets was chosen for further construction of the artificial miRNA.

### Vectors and cloning procedure

The vector pKDSR(I) was created by converting the unique *Asc*I of pRedroot [35] to a *Pac*I site, in a way that the gene with its promoter and terminator was flanked by two *Pac*I sites. Likewise, a *Kpn*I site of pK7GWIWG2(I) [36] was converted into a *Pac*I site. The *Pac*I flanked cassette was excised from pRedroot and ligated into the novel *Pac*I site of pK7GWIWG2(I). The resulting vector was named pKDSR(I).

For the construction of the pRed-amiR vectors, the final amplification product of the overlapping PCR was first cloned into pCR2.1 (Invitrogen). The PCR product included parts of the pBluescript II SK (+) multiple cloning site. The amiR precursor molecule was removed from the pCR2.1 vector by using the *Spe*I and *Mlu*I restriction sites and ligated into the appropriate sites of pRed vectors. Final pRed-amiR constructs were sequenced and used for root transformation (pRed-35Spro::amiR-*dsred*, pRed-MtPt4pro::amiR-*MtErf*1) as described recently [32]. Additionally, for tobacco leaf infiltration assays, the amiR-*dsred* precursor molecule was inserted *via Not*I and *Kpn*I sites into pORE-E4 [24].

### Overlapping PCR using the miR159b backbone to design an artificial miRNA

The miR159b backbone was synthesized by gene synthesis (MWG) and an additional MluI site was added to the 3′ end of the miR precursor. This molecule was cloned into the *SpeI* and *PstI* restriction site of pBluescript II SK (+). The resulting pB159b vector represents the template for the overlapping PCR to generate amiR precursor molecules. For this purpose four different primers are designed according to [37].

Primer I: GTX_1_…X_21_AAATTGGACACGCGTct (X_1_-X_21_ are the designed amiR sequence).

Primer II: TTY_1_…Y_21_ACAAAAAGATCAAGGC (Y_1_-Y_21_ are the amiR sequence in reverse complement orientation).

Primer III: TTZ_1_…Z_21_TCTAAAAGGAGGTGATAG (Z_1_-Z_21_ are the amiR sequence in reverse complement orientation, with the exception that Z_11_ and Z_12_ have to be modified to not pair (also no G:U non-Watson-Crick pairing) to position X_10_ and X_11_, respectively. Also Z_21_ has to be changed to not pair to X_1_.

Primer IV: GAN_1_…N_21_ AATTAGGTTactagt (N_1_-N_21_ are reverse complement of Z_1_-Z_21_). Primer sequences used to create amiRDsRED and amiR*MtErf1* are given in the Additional file 2: Table S1.

Three independent PCRs were performed with pB159b as template and the following three primer combinations (1) primer A + primer I, (2) primer II + primer III, (3) primer B + primer IV. The PCR products were loaded into a single well of an 2% agarose gel followed by gel purification. The resulting mixture of products was used as a template for a final PCR using primer A + primer B. The single PCR product was subcloned into pCR2.1 (Invitrogen) and sequenced to check for a correct amiR precursor sequence and a stem-loop folding identical to mtr-miR159b.

### RNA extraction, RT-PCR and quantitative RT-PCR

Total RNA was extracted from liquid nitrogen frozen and ground tissue using the miRVana miRNA extraction Kit (Ambion) and Plant Isolation Aid step (Ambion) according to the manufacturer’s instructions. All PCRs were carried out as described earlier in [23,38].

### Protein extraction and Western blotting

Frozen plant tissue was ground and proteins were extracted with rigorous vortexing in 4 ml of homogenization buffer per gram fresh weight. The homogenization buffer consisted of 100 mM HEPES pH 7.5, 10% glycerol, 5 mM DTT, cOmplete ULTRA tablet – EDTA free (Roche) proteinase inhibitor cocktail. The extract was centrifuged at 14000 g for 15 min (4°C) and the supernatant was collected and aliquots were frozen at −20°C. The protein concentration was determined using QuickStart Bradford Protein Assay (Biorad) with the provided γ-globulin standard following the manufacturer’s instructions using the microtiter plate protocol.

For the western blot analysis an equal amount of 15 μg of protein from extracts of tobacco plants and 10 μg of proteins from Medicago root extracts were resolved on separately on 1 mm 12% SDS-PAGE mini-gels, respectively, and subsequently transferred onto Immobilon-P PVDF membranes (Millipore) by semi-dry blotting according to Immobilon-P transfer membrane user guide. Detection of DsRed was carried out with primary antibodys using 0.4 μg/ml (1:1250) rabbit anti-RFP tag antibody (GenScript) mixed with rabbit anti-RubisCO activase (Agrisera) in a 1:10000 dilution, the latter serving to detect RubisCO activase as a loading control. The secondary goat anti-rabbit antibody conjugated with alkaline phosphatase (Abcam) was used as a 1:10000 dilution. The protein bands were visualized using NBT/BCIP (Roche). The specific proteins were identified by comparison to a pre-stained protein size marker (Thermo scientific).

### Statistics

To test for difference between plant genotype and treatment, data were analyzed by Student’s *t*-test for pairwise comparisons using the Sigmaplot software package (Systat, Germany).

## Competing interest

The authors declare no competing interests.

## Authors’ contributions

EAD and JT carried out the molecular analyses, participated in the design of the study, performed the statistical analysis and drafted the manuscript. AR carried out the promoter reporter assays. NG carried out the stable plant transformation. FK conceived of the study, and participated in its design and coordination and helped to draft the manuscript. All authors read and approved the final manuscript.

## Supplementary Material

Additional file 1: Figure S1 Sequence of the mtr-miR159b backbone for amiRNA expression in pBluescriptII SK+ vector. AmiRNA constructs are generated from this template using an overlapping PCR strategy (Schwab *et al*., 2006; Ossowski *et al*., 2008; Schwab *et al*., 2010). An additional *Mlu*I restriction site was introduced for subcloning into the pRed vectors. **Figure S2.** Schematic representation of the T-DNA of the pRed vector series. Vectors are available as expression vectors (amiR expression) or RNAi vectors. An ubiquitin10 (ubi10) promoter-driven dsred gene allows the identification of transformed roots via the dsred fluorescence. The MtPt4 promoter mediates a strong expression in arbuscule-containing cells (pRed-PT4). Alternatively, the ubiquitin10 (pRed-ubi3 ) or 2×35S (pRed-35S) promoter mediate a constitutive expression the corresponding constructs. TL/R: left/right border of T-DNA, pro: promoter, nptII: neomycin phosphotransferase (kanamycin resistance) ter: terminator, OCS: Octopin synthase. **Figure S3.** Activity of the MtErf1 promoter in arbuscule-containing cells of mycorrhizal roots of *M. truncatula*. 1145 bp *MtErf*1 promoter region were fused to cyan fluorescent protein (CFP). and transferred by *Agrobacterium rhizogenes* transformation into *M. truncatula* roots. Roots were colonized with *R. irregularis*. Three weeks after inoculation, roots were harvested and CFP fluorescence was observed in cross section. Fungal structures were stained with WGA- Alexafluor 594. Arrowheads indicate arbuscules, iH: intercellular hyphae, P: pericycle, scale bars indicate 25 μm. **Figure S4.** Alignments of MtERF1 wit homologs from *Glycine max* Gm_1 (XP_003533548.1), Gm_2 (XP_003551723.1), Gm_3 (XP_003530686.1), Gm_4 (XP_003553203.1), *Populus trichocarpa* Pt_1 (XP_002323836.1 ), Pt_2 (XP_002315794.1 ), *Ricinus communis* Rc_1 (XP_002517474.1 ), *Vitis vinifera* Vv_1(CAN79925.1), Vv_2 (XP_003633849.1), Vv_3 (XP_002270149.1 ), *Fragaria vesca* Fv_1(XP_004304943.1 ), Fv_2 (XP_004298447.1 ), *Prunus persica* Pp_1 (EMJ17977.1 ), Pp_2 (EMJ18018.1 ), Pp_3 (EMJ27523.1 ), *Cucumis sativus* Cs_1 (XP_004147491.1 ). The two AP2 domains are underlined in red and identical positions are highlighted in grey. Click here for file

Additional file 2: Table S1 Primer and amiR sequences used in this study. Click here for file
