# Supplementary material for: An endogenous artificial microRNA system for unraveling the function of root endosymbioses related genes in Medicago truncatula
Source: BMC Plant Biol. 2013 May 16;13:82. doi: 10.1186/1471-2229-13-82 (PMC3679836; doi:10.1186/1471-2229-13-82)
Supplement: Additional file 1: Figure S1 — Sequence of the mtr-miR159b backbone for amiRNA expression in pBluescriptII SK+ vector. AmiRNA constructs are generated from this template using an overlapping PCR strategy (Schwab et al., 2006; Ossowski et al., 2008; Schwab et al., 2010). An additional MluI restriction site was introduced for subcloning into the pRed vectors. Figure S2. Schematic representation of the T-DNA of the pRed vector series. Vectors are available as expression vectors (amiR expression) or RNAi vectors. An ubiquitin10 (ubi10) promoter-driven dsred gene allows the identification of transformed roots via the dsred fluorescence. The MtPt4 promoter mediates a strong expression in arbuscule-containing cells (pRed-PT4). Alternatively, the ubiquitin10 (pRed-ubi3 ) or 2×35S (pRed-35S) promoter mediate a constitutive expression the corresponding constructs. TL/R: left/right border of T-DNA, pro: promoter, nptII: neomycin phosphotransferase (kanamycin resistance) ter: terminator, OCS: Octopin synthase. Figure S3. Activity of the MtErf1 promoter in arbuscule-containing cells of mycorrhizal roots of M. truncatula. 1145 bp MtErf1 promoter region were fused to cyan fluorescent protein (CFP). and transferred by Agrobacterium rhizogenes transformation into M. truncatula roots. Roots were colonized with R. irregularis. Three weeks after inoculation, roots were harvested and CFP fluorescence was observed in cross section. Fungal structures were stained with WGA- Alexafluor 594. Arrowheads indicate arbuscules, iH: intercellular hyphae, P: pericycle, scale bars indicate 25 μm. Figure S4. Alignments of MtERF1 wit homologs from Glycine max Gm_1 (XP_003533548.1), Gm_2 (XP_003551723.1), Gm_3 (XP_003530686.1), Gm_4 (XP_003553203.1), Populus trichocarpa Pt_1 (XP_002323836.1 ), Pt_2 (XP_002315794.1 ), Ricinus communis Rc_1 (XP_002517474.1 ), Vitis vinifera Vv_1(CAN79925.1), Vv_2 (XP_003633849.1), Vv_3 (XP_002270149.1 ), Fragaria vesca Fv_1(XP_004304943.1 ), Fv_2 (XP_004298447.1 ), Prunus persica Pp_1 (EMJ17977.1 ), P [file 1471-2229-13-82-S1.pptx]

## Slide 1
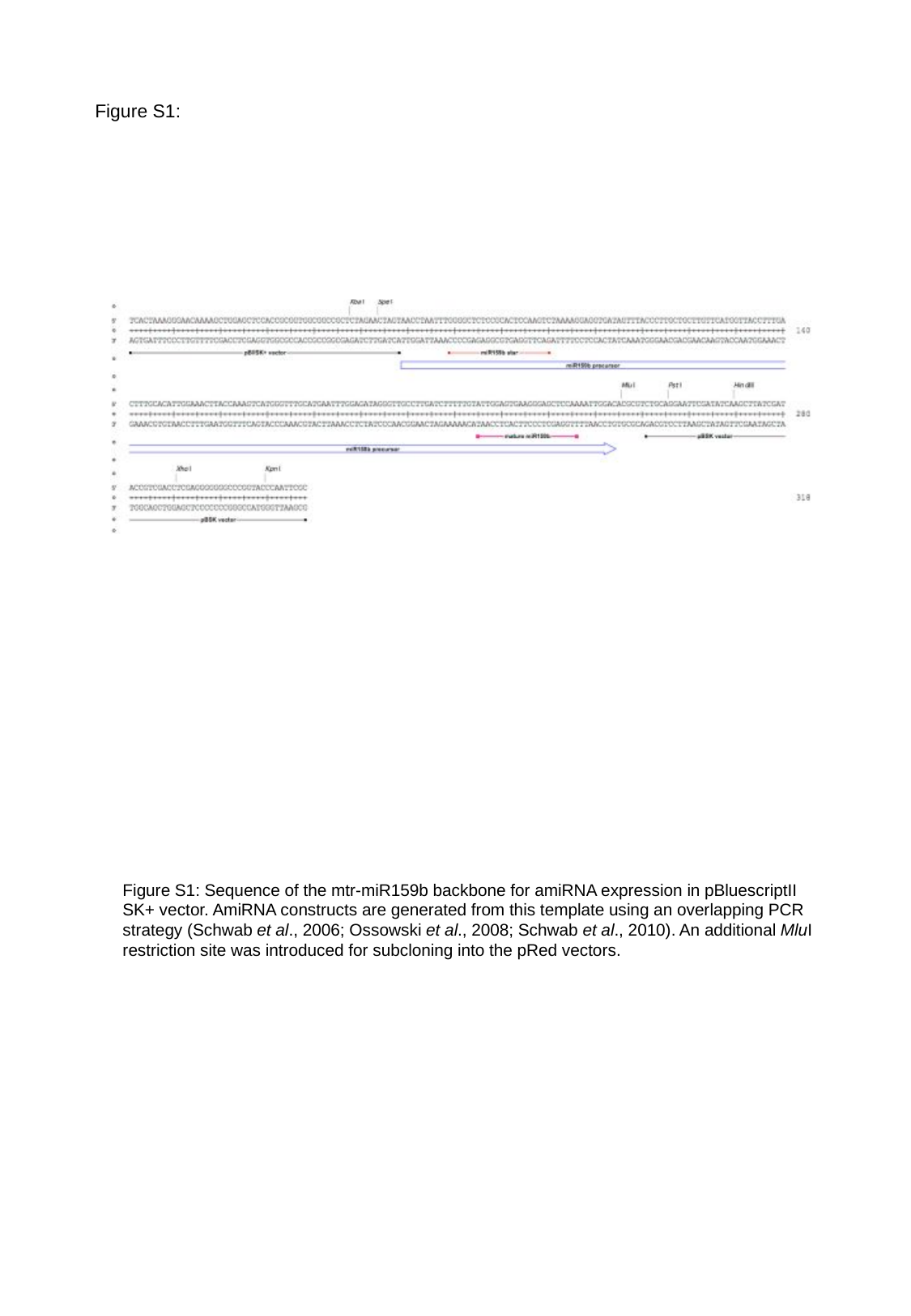

Figure S1:
Figure S1: Sequence of the mtr-miR159b backbone for amiRNA expression in pBluescriptII SK+ vector. AmiRNA constructs are generated from this template using an overlapping PCR strategy (Schwab et al., 2006; Ossowski et al., 2008; Schwab et al., 2010). An additional MluI restriction site was introduced for subcloning into the pRed vectors.

## Slide 2
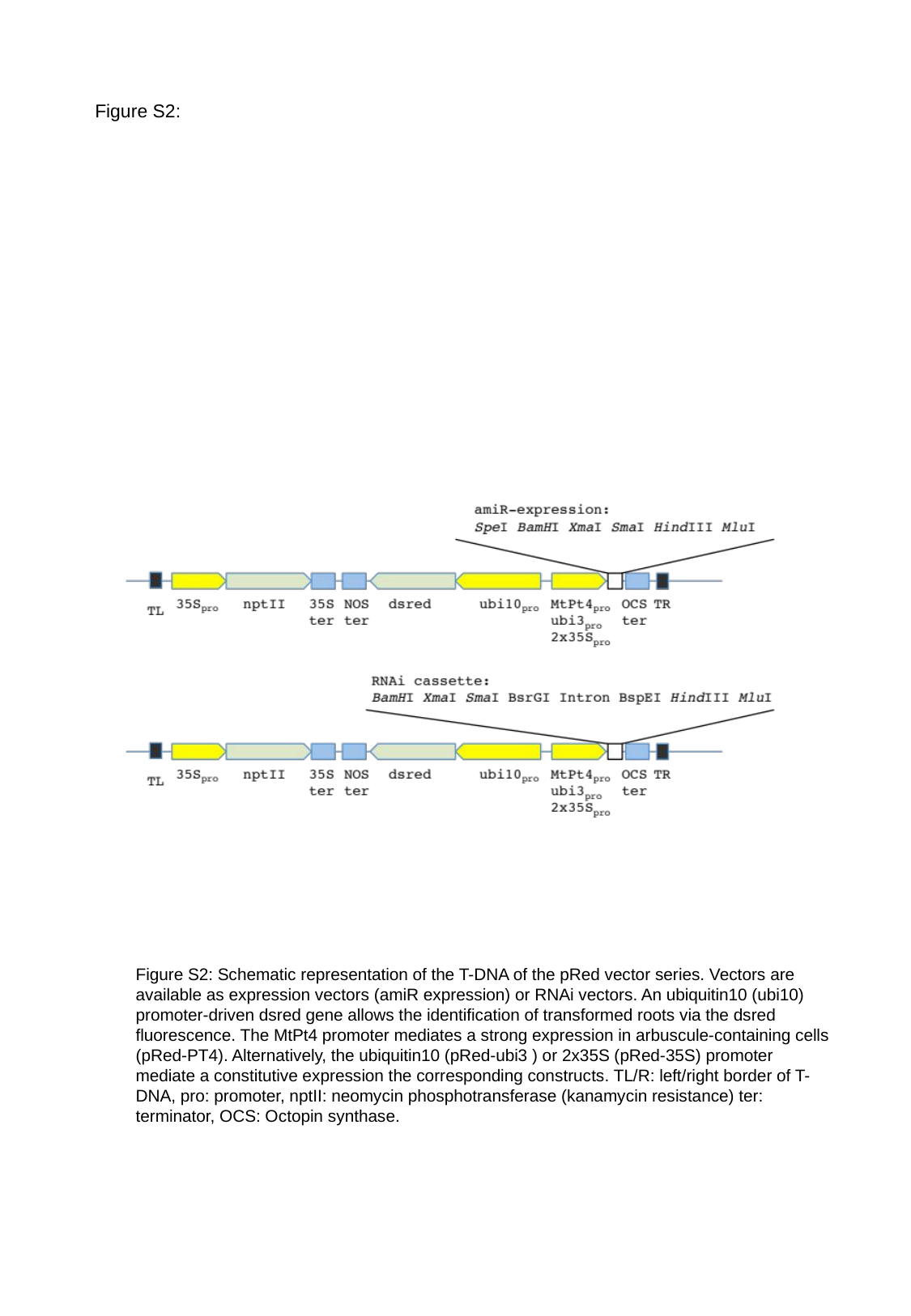

Figure S2:
Figure S2: Schematic representation of the T-DNA of the pRed vector series. Vectors are available as expression vectors (amiR expression) or RNAi vectors. An ubiquitin10 (ubi10) promoter-driven dsred gene allows the identification of transformed roots via the dsred fluorescence. The MtPt4 promoter mediates a strong expression in arbuscule-containing cells (pRed-PT4). Alternatively, the ubiquitin10 (pRed-ubi3 ) or 2x35S (pRed-35S) promoter mediate a constitutive expression the corresponding constructs. TL/R: left/right border of T-DNA, pro: promoter, nptII: neomycin phosphotransferase (kanamycin resistance) ter: terminator, OCS: Octopin synthase.

## Slide 3
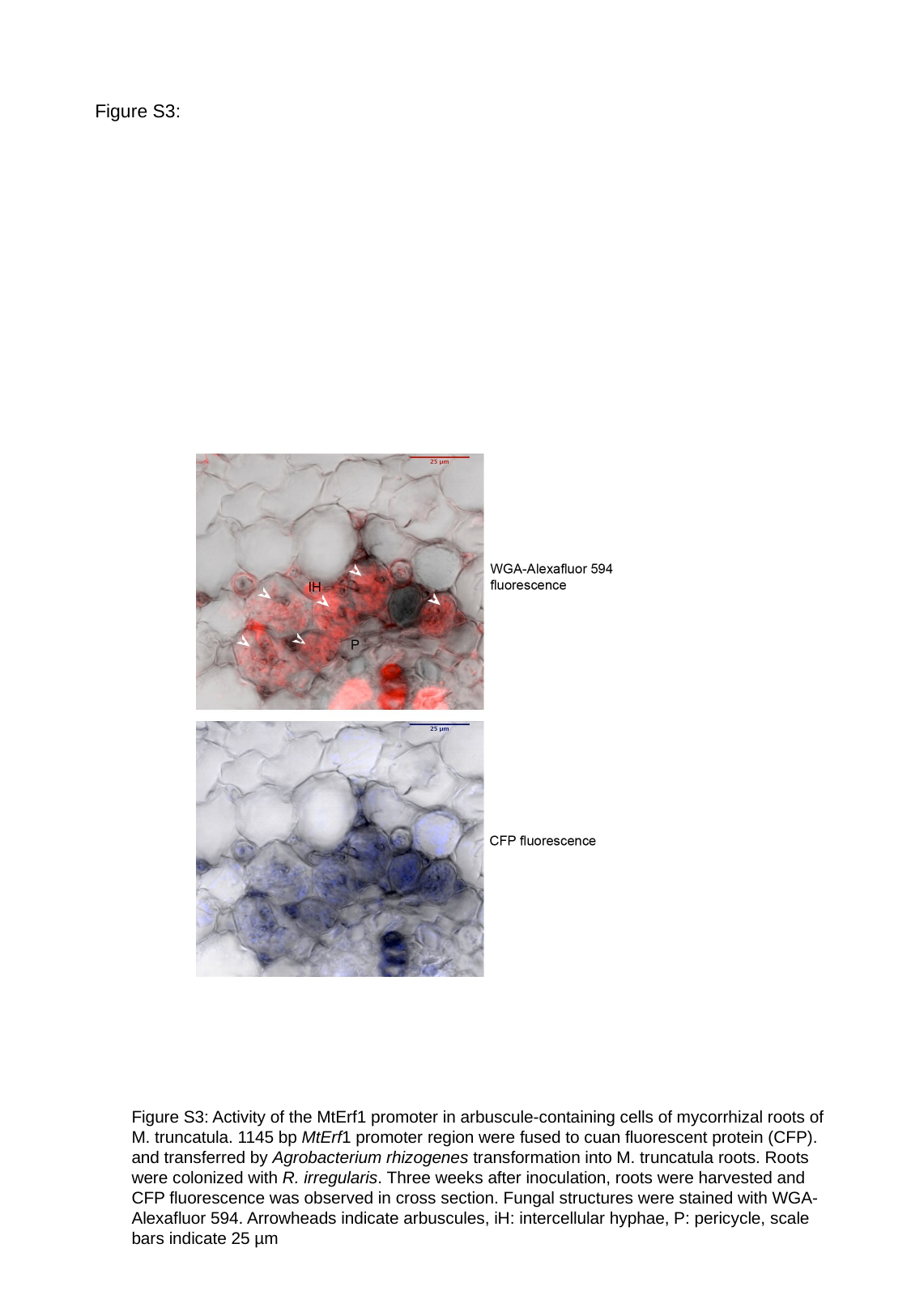

Figure S3:
Figure S3: Activity of the MtErf1 promoter in arbuscule-containing cells of mycorrhizal roots of M. truncatula. 1145 bp MtErf1 promoter region were fused to cuan fluorescent protein (CFP). and transferred by Agrobacterium rhizogenes transformation into M. truncatula roots. Roots were colonized with R. irregularis. Three weeks after inoculation, roots were harvested and CFP fluorescence was observed in cross section. Fungal structures were stained with WGA- Alexafluor 594. Arrowheads indicate arbuscules, iH: intercellular hyphae, P: pericycle, scale bars indicate 25 µm

## Slide 4
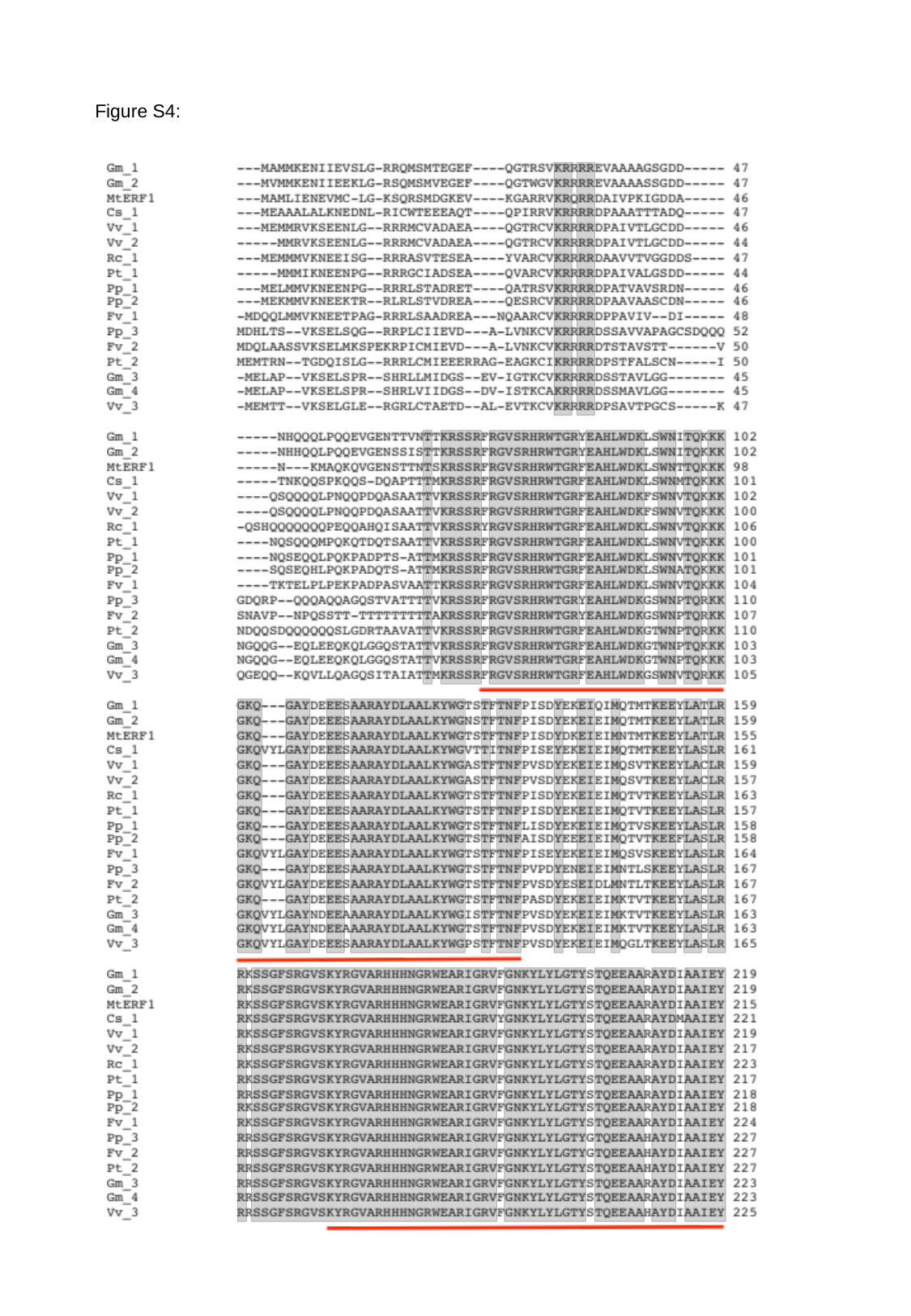

Figure S4:

## Slide 5
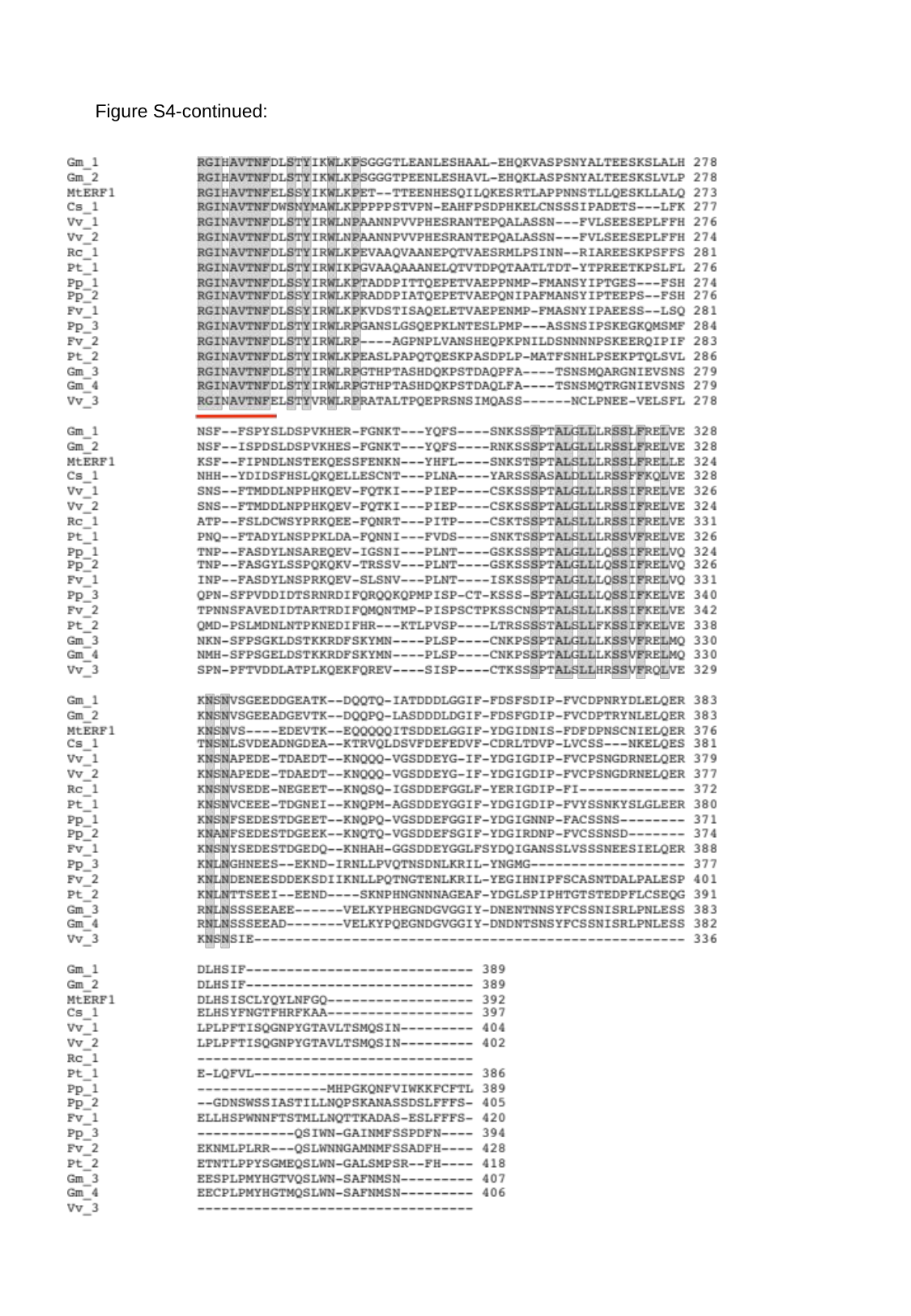

Figure S4-continued:

## Slide 6
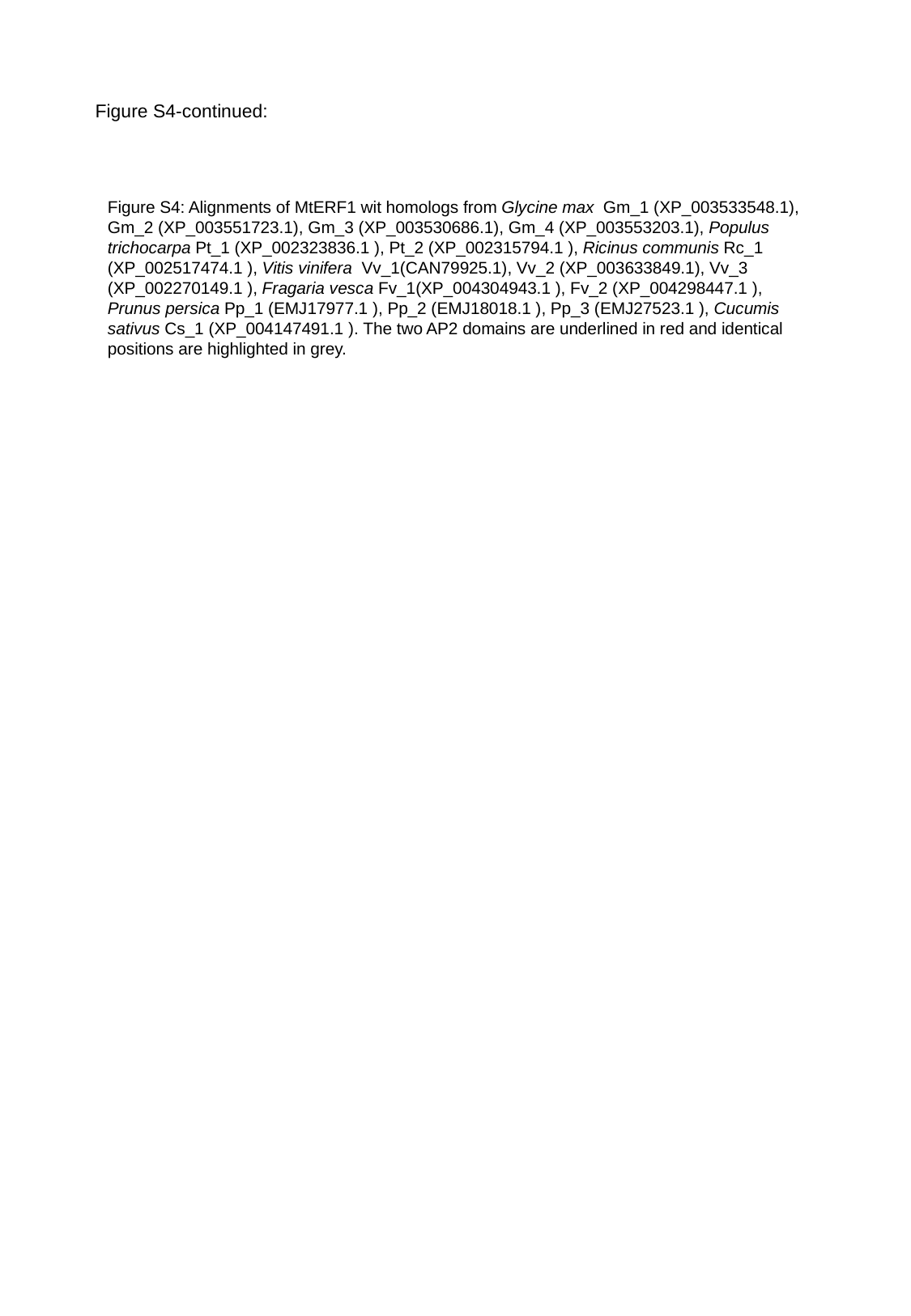

Figure S4-continued:
Figure S4: Alignments of MtERF1 wit homologs from Glycine max Gm_1 (XP_003533548.1), Gm_2 (XP_003551723.1), Gm_3 (XP_003530686.1), Gm_4 (XP_003553203.1), Populus trichocarpa Pt_1 (XP_002323836.1 ), Pt_2 (XP_002315794.1 ), Ricinus communis Rc_1 (XP_002517474.1 ), Vitis vinifera Vv_1(CAN79925.1), Vv_2 (XP_003633849.1), Vv_3 (XP_002270149.1 ), Fragaria vesca Fv_1(XP_004304943.1 ), Fv_2 (XP_004298447.1 ), Prunus persica Pp_1 (EMJ17977.1 ), Pp_2 (EMJ18018.1 ), Pp_3 (EMJ27523.1 ), Cucumis sativus Cs_1 (XP_004147491.1 ). The two AP2 domains are underlined in red and identical positions are highlighted in grey.
