# Supplementary material for: An endogenous artificial microRNA system for unraveling the function of root endosymbioses related genes in Medicago truncatula
Source: BMC Plant Biol. 2013 May 16;13:82. doi: 10.1186/1471-2229-13-82 (PMC3679836; doi:10.1186/1471-2229-13-82)
Supplement: Additional file 2: Table S1 — Primer and amiR sequences used in this study. [file 1471-2229-13-82-S2.docx]

| **target** | **Forward primer (5´-3´)** | **Reverse primer (5´-3´)** |
| --- | --- | --- |
| *MtEf*1 | GACAAGCGTGTGATTGAGAG | TTTCATCATACCTGCTCTTGGA |
| *MtErf*1 | TGGCACAGAAACAAGTTGGA | CTCTGTACTCAATTGCAGCT |
| MtErf1pro | CACCAAATGCAAATGACAACGA | TTTGTTCCTAATTCTTCTCTACTTAG |
| *dsRED* | CCAAGAACGTCATCAAGGAG | CCCTTGGTCACCTTCAG |
| *MtPt*4 | CAAGAAAGATTAGACGCGCAA | GTTTCCGTCACCAAGAACGTG |
| *RiEf* | TGTTGCTTTCGTCCCAAT | GGTTTATCGGTAGGTCGA |
| mature amiRdsred | ATCCGTACGTAGTAGTAGCC | CCAGTGCAGGGTCCGAGGT |
| *npt*II | CGCCAGCCGAACTGTTCG | CGATACCGTAAAGCACGAGGAAGC |

**amiR-*dsRed* stem-loop RT primer:** GTCGTATCCAGTGCAGGGTCCGAGGTATTCGCACTGGATACGACCTGCCC

**The following primers are used for amiR construction:**

**Primer A** CTGCAAGGCGATTAAGTTGGGTAAC

**Primer B** GCGGATAACAATTTCACACAG

**amiR-*dsred*:** UACGUAGUAGUAGCCGGGCAG

**amiR-*dsRed* overlapping PCR:**

primer I: GTTACGTAGTAGTAGCCGGGCAGAAATTGGACACGCGTCT

primer II: TTCTGCCCGGCTACTACTACGTAACAAAAAGATCAAGGC

primer III: TTCTGCCCGGCTTGTACTACGTCTCTAAAAGGAGGTGATA

primer IV: GAGACGTAGTACAAGCCGGGCAGAATTAGGTTACTAGT

**amiR-*MtErf*1:** UCUUGUGUACUAUAGGUUCGA

**amiR-*MtErf1* overlapping PCR:**

primer I: GTTCTTGTGTACTATAGGTTCGAAAATTGGACACGCGTCT

primer II: TTTCGAACCTATAGTACACAAGAACAAAAAGATCAAGGC

primer III: TTTCGAACCTATCTTACACAAGCTCTAAAAGGAGGTGATAG

primer IV: GAGCTTGTGTAAGATAGGTTCGAAATTAGGTTACTAGT
